# Neutrophil Count and Neutrophil-to-Lymphocyte Ratio Predict Rheumatic Heart Disease in Children With Acute Rheumatic Fever

**DOI:** 10.7759/cureus.106217

**Published:** 2026-03-31

**Authors:** Hülya Akat, Halil Keskin, Fuat Laloglu, Esra Laloglu, Muhammet A Güler, Naci Ceviz

**Affiliations:** 1 Pediatrics, Atatürk University Faculty of Medicine, Erzurum, TUR; 2 Pediatric Infectious Diseases, Ankara University Faculty of Medicine, Ankara, TUR; 3 Pediatric Cardiology, Atatürk University Faculty of Medicine, Erzurum, TUR; 4 Medical Biochemistry, Atatürk University Faculty of Medicine, Erzurum, TUR; 5 Pediatric Nephrology, Atatürk University Faculty of Medicine, Erzurum, TUR; 6 Pediatric Nephrology, Erzurum City Hospital, Erzurum, TUR

**Keywords:** acute rheumatic fever, inflammation, neutrophil count, neutrophil-to-lymphocyte ratio, rheumatic heart disease

## Abstract

Background: Acute rheumatic fever (ARF) remains an important cause of acquired heart disease in children, particularly in low- and middle-income countries. Identifying simple and accessible inflammatory markers that reflect disease activity or predict cardiac involvement may improve clinical evaluation. This study aimed to evaluate neutrophil count, neutrophil-to-lymphocyte ratio (NLR), and mean platelet volume (MPV) in pediatric patients with ARF and to assess their potential predictive value for the development of rheumatic heart disease (RHD).

Methods: This retrospective study included pediatric patients diagnosed with ARF between June 2015 and July 2020. Clinical, laboratory, and echocardiographic data were obtained from medical records. Hematological parameters, including neutrophil count, lymphocyte count, MPV, and platelet-large cell ratio (P-LCR), were analyzed and compared with those of healthy controls. Patients were followed during routine clinical follow-up to evaluate the development of RHD. Receiver operating characteristic (ROC) curve analysis was performed to assess the predictive value of neutrophil count and NLR.

Results: A total of 56 children with ARF and 35 healthy controls were included. Neutrophil count and NLR were significantly higher in patients compared to controls, whereas platelet indices did not differ significantly. A positive correlation was observed between NLR and C-reactive protein (CRP) levels. During follow-up, higher neutrophil counts at diagnosis were associated with persistent valvular involvement. ROC analysis demonstrated moderate predictive performance for neutrophil count (area under the curve (AUC) = 0.72) and NLR (AUC = 0.68) in predicting the development of RHD.

Conclusions: Neutrophil count and NLR may reflect inflammatory activity in children with ARF and may help identify patients at risk for developing RHD. However, their predictive value appears to be moderate and should be interpreted with caution. These findings are preliminary and hypothesis-generating, and further prospective studies are needed to confirm these results.

## Introduction

Acute rheumatic fever (ARF) is an immune-mediated inflammatory disorder that develops as a delayed complication of infection with group A β-hemolytic Streptococci [1,2]. Despite improvements in healthcare systems, ARF remains an important cause of acquired heart disease in children worldwide, particularly in low- and middle-income countries where the burden of rheumatic heart disease (RHD) remains substantial [3-7]. Cardiac involvement is the most critical manifestation of the disease, as persistent valvular damage may eventually lead to chronic RHD and long-term morbidity.

Early identification of patients who are at increased risk of developing RHD is, therefore, clinically important. In recent years, several inflammatory markers derived from routine laboratory tests have been investigated as potential indicators of disease activity and prognosis in cardiovascular and inflammatory diseases [8-13]. These markers may provide useful insight into the degree of systemic inflammation and may contribute to clinical decision-making.

Mean platelet volume (MPV) is considered an indicator of platelet activation and production dynamics, while the neutrophil-to-lymphocyte ratio (NLR), which can be easily calculated from a complete blood count, reflects the balance between inflammatory and immune regulatory responses [14,15]. Previous studies have suggested that NLR may serve as a simple and inexpensive marker of systemic inflammation and may be associated with disease severity and cardiovascular outcomes in various clinical settings [9,16,17].

However, evidence regarding the relationship between platelet indices, NLR, and the development of RHD in children with ARF remains limited [18-21]. Therefore, the present study aimed to evaluate neutrophil count, NLR, and MPV in pediatric patients with ARF and to assess their predictive value for the development of RHD during follow-up.

## Materials and methods

This retrospective study was conducted at the Pediatric Cardiology Outpatient Clinic of Atatürk University Faculty of Medicine. The study aimed to evaluate inflammatory hematological markers in ARF and to investigate their predictive value for the development of RHD during follow-up. The medical records of pediatric patients diagnosed with ARF between June 2015 and July 2020 were reviewed. The diagnosis of ARF was established according to the revised Jones Criteria recommended by the American Heart Association [2]. Clinical, laboratory, and echocardiographic findings of the patients were obtained from hospital archive records.

Complete blood count parameters obtained at the time of diagnosis and after completion of anti-inflammatory treatment during follow-up were evaluated. Hematological parameters including neutrophil count, lymphocyte count, MPV, and platelet-large cell ratio (P-LCR) were recorded. The NLR was calculated by dividing the absolute neutrophil count by the lymphocyte count. In addition, inflammatory markers including erythrocyte sedimentation rate (ESR) and C-reactive protein (CRP) levels were recorded when available.

Patients with incomplete medical records, hematological diseases, chronic systemic diseases, or active infections other than Streptococcal infection were excluded from the study. A control group consisting of age- and sex-matched healthy children without acute or chronic inflammatory diseases was included for comparison.

Echocardiographic findings were reviewed to determine the presence of carditis and valvular involvement. Patients were followed during clinical follow-up to evaluate the persistence of valvular lesions and the development of RHD. Follow-up echocardiographic evaluations were performed during routine outpatient visits. Persistent valvular involvement was defined as the presence of valvular regurgitation on follow-up echocardiography, and the primary outcome of the study was defined as the development of RHD during follow-up. Patients with missing follow-up echocardiographic data were excluded from analyses evaluating the development of RHD.

Statistical analyses were performed using the Statistical Package for the Social Sciences (SPSS) version 20. The normal distribution of continuous variables was evaluated using visual methods (histograms and probability plots) and analytical tests including the Kolmogorov-Smirnov and Shapiro-Wilk tests. Continuous variables were expressed as mean ± SD or median (interquartile range) where appropriate, and categorical variables were expressed as numbers and percentages.

For comparisons between groups, parametric tests (Student’s t-test and analysis of variance (ANOVA)) were used for normally distributed variables, while non-parametric tests (Mann-Whitney U test, Kruskal-Wallis test, and Wilcoxon test) were used for variables that did not follow normal distribution. Gender distribution between groups was compared using the chi-square test. Correlations between continuous variables were evaluated using Spearman’s correlation analysis. Receiver operating characteristic (ROC) curve analysis was performed to determine the sensitivity and specificity of neutrophil count at diagnosis in predicting the development of RHD. A p-value of less than 0.05 was considered statistically significant.

Ethical approval

This study was approved by the Ethics Committee of Atatürk University Faculty of Medicine (Date: October 1, 2020, Meeting No: 08, Decision No: 60). Because of the retrospective design of the study and the use of existing medical records, the requirement for informed consent was waived.

## Results

A total of 56 pediatric patients diagnosed with ARF during the study period were included in the study. An age- and sex-matched healthy control group consisting of 35 children was also included for comparison. The demographic characteristics of the study population are summarized in Table 1.

**Table 1:** Demographic characteristics of the patient and control groups Values are expressed as mean ± SD or number. Continuous variables were compared using Student’s t-test and categorical variables using the chi-square test.

Parameter	Patient group (n=56) Mean ± SD	Control group (n=35) Mean ± SD	Test statistic	p
Age (years)	11.8 ± 3.5	10.8 ± 2.84	t = 1.40	0.165
Sex (Male/Female)	26 / 30	13 / 22	χ² = 0.42	0.514

Complete blood count parameters of patients with ARF were compared with those of the control group. Neutrophil counts and NLR values were significantly higher in patients with ARF compared with the control group, whereas lymphocyte counts were similar between the groups. Platelet indices, including MPV and P-LCR, did not differ significantly between patients and controls (Table 2).

**Table 2:** Laboratory parameters of the patient and control groups Paired t-test was used for comparisons between admission and post-treatment values (I–II). One-way ANOVA was used for comparisons among admission, post-treatment, and control groups (I–II–III). MPV: Mean platelet volume; P-LCR: Platelet-large cell ratio; NLR: Neutrophil-to-lymphocyte ratio; ANOVA: Analysis of variance

Parameter	Patient admission (I) n=56	Patient after treatment (II) n=56	Control group (III) n=35	F	p (I-II-III)	p (I-II)	p (I-III)	p (II-III)
MPV (fL)	9.4 ± 0.8	9.7 ± 0.7	9.53 ± 0.66	0.65	0.521	0.19	0.42	0.63
P-LCR (%)	20.3 ± 5.9	22.1 ± 6.13	21.2 ± 5.47	2.1	0.349	0.21	0.52	0.67
Neutrophil count	7494.9 ± 3203.9	4397.4 ± 1771.6	3501.1 ± 1368.6	52.2	<0.001	<0.001	<0.001	0.021
Lymphocyte count	2553.3 ± 866.3	3046.3 ± 1001.2	2797.1 ± 849.7	7.3	0.027	0.008	0.123	0.433
NLR	3.34 ± 1.94	1.58 ± 0.84	1.41 ± 1.09	47.4	<0.001	<0.001	<0.001	0.05

Laboratory parameters were also evaluated before and after anti-inflammatory treatment in patients with ARF. No significant differences were observed in platelet indices between the acute phase and the post-treatment period. However, inflammatory markers and neutrophil counts showed significant changes consistent with disease activity (Table 2).

Patients were further analyzed according to the presence or absence of carditis. Subgroup analysis based on the presence of carditis was performed to evaluate whether hematological inflammatory markers differed between patients with and without cardiac involvement. Comparisons between patients with and without carditis did not reveal significant differences in platelet indices. Similarly, neutrophil and lymphocyte counts were not significantly different between the two groups (Table 3).

**Table 3:** Laboratory parameters according to presence of carditis Values are expressed as mean ± SD. Comparisons among groups were performed using one-way ANOVA. MPV: Mean platelet volume; P-LCR: Platelet-large cell ratio; NLR: Neutrophil-to-lymphocyte ratio; ANOVA: Analysis of variance

Parameter	Carditis (n=46)	No carditis (n=10)	Control (n=35)	F	p
MPV	9.48 ± 0.75	9.2 ± 0.94	9.53 ± 0.66	0.97	0.616
P-LCR	20.7 ± 5.6	18.5 ± 7.11	21.2 ± 5.47	1.18	0.554
Neutrophil count	7142 ± 2917	9083 ± 4069	3501 ± 1368	41.34	<0.001
Lymphocyte count	2502 ± 930	2784 ± 443	2797 ± 849	4.63	0.099
NLR	3.3 ± 1.92	3.53 ± 2.12	1.41 ± 1.09	33.91	<0.001

Correlation analysis demonstrated a positive correlation between NLR and CRP levels, suggesting an association between systemic inflammation and hemogram parameters (Table 4).

**Table 4:** Correlation between hemogram parameters and inflammatory markers Spearman's correlation analysis was used. MPV: Mean platelet volume; P-LCR: Platelet-large cell ratio; NLR: Neutrophil-to-lymphocyte ratio; ESR: Erythrocyte sedimentation rate; CRP: C-reative protein

Parameter	ESR (r)	p	CRP (r)	p
MPV	0.104	0.431	−0.050	0.721
P-LCR	0.162	0.224	−0.015	0.915
Neutrophil count	0.237	0.071	0.222	0.107
Lymphocyte count	0.159	0.228	−0.226	0.1
NLR	0.19	0.149	0.345	0.01

During follow-up, patients were evaluated for persistence of valvular lesions. Follow-up echocardiographic data were available for only 20 patients; therefore, analyses evaluating the development of RHD were performed in this subgroup of patients. The mean neutrophil count at the time of diagnosis was significantly higher in patients with persistent valvular involvement compared with those without valvular lesions (Table 5).

**Table 5:** Admission laboratory parameters according to development of RHD Values are expressed as mean ± SD. Comparisons between groups were performed using Student’s t-test. MPV: Mean platelet volume; P-LCR: Platelet-large cell ratio; NLR: Neutrophil-to-lymphocyte ratio; RHD: Rheumatic heart disease

Parameter	Lesion (+) n=13	Lesion (−) n=7	Test statistic	p
MPV	9.6 ± 0.82	9.5 ± 0.83	t = 0.34	0.739
PLCR	21.6 ± 6.7	20.6 ± 6.9	t = 0.29	0.771
Neutrophil count	7770 ± 2387	5045 ± 2787	t = 2.23	0.037
Lymphocyte count	2495 ± 883	2497 ± 852	t = −0.003	0.998
NLR	3.5 ± 1.8	2.2 ± 1.4	t = 1.67	0.107

ROC curve analysis was performed to evaluate the predictive value of admission neutrophil count and NLR for the development of RHD during follow-up. Neutrophil count demonstrated a moderate predictive performance (area under the curve (AUC) = 0.72, 95% confidence interval (CI): 0.54-0.89, p = 0.037). A cut-off value of 6,500 cells/µL yielded a sensitivity of 69.2% and a specificity of 71.4%.

**Figure 1:**
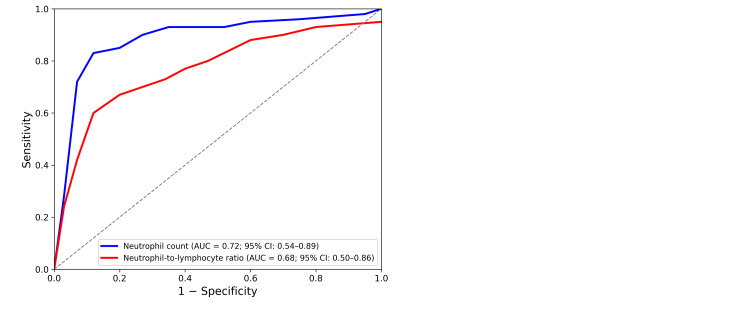
ROC curves of neutrophil count and NLR for predicting the development of RHD ROC: Receiver operating characteristic; NLR: Neutrophil-to-lymphocyte ratio; RHD: Rheumatic heart disease

Similarly, the NLR also showed moderate predictive ability (AUC = 0.68, 95% CI: 0.50-0.86, p = 0.048). The optimal cut-off value for NLR was 2.8, with a sensitivity of 61.5% and specificity of 71.4% (Table 6, Figure 1).

**Table 6:** ROC analysis of neutrophil count and NLR for predicting RHD during follow-up ROC curve analysis was performed to evaluate the predictive value of admission neutrophil count and NLR for the development of RHD during follow-up. NLR: Neutrophil-to-lymphocyte ratio; AUC: Area under the curve; CI: Confidence interval; ROC: Receiver operating characteristic; RHD: Rheumatic heart disease

Parameter	AUC (95% CI)	Cut-off value	Sensitivity (%)	Specificity (%)	p
Neutrophil count	0.72 (0.54–0.89)	6500 cells/µL	69.2	71.4	0.037
NLR	0.68 (0.50–0.86)	2.8	61.5	71.4	0.048

## Discussion

ARF remains an important cause of acquired cardiovascular disease in children, particularly in developing countries where Streptococcal infections remain prevalent [1,3,5]. Early identification of laboratory parameters that may reflect inflammatory activity or predict cardiac involvement is therefore clinically important.

Our findings demonstrated that neutrophil counts and NLR values were significantly higher in children with ARF compared with healthy controls. These results are consistent with previous studies suggesting that NLR may reflect systemic inflammatory response and may serve as a useful marker of inflammatory activity in cardiovascular and inflammatory diseases [8-13]. Neutrophils are key components of the innate immune response and play an important role in acute inflammatory processes.

In contrast, platelet indices including MPV and P-LCR were not significantly different between patients and controls in our study. Although platelet indices have been proposed as potential inflammatory markers in various cardiovascular and inflammatory conditions [14,22,23], our findings suggest that these parameters may not be reliable indicators of inflammatory activity in children with ARF.

Subgroup analysis according to the presence of carditis was performed to determine whether hematological inflammatory markers differed in patients with cardiac involvement. Because carditis is the most important clinical manifestation of ARF and is closely associated with the development of RHS [6,7,24,25], evaluating laboratory parameters according to cardiac involvement may provide additional insight.

Another important finding of our study was that neutrophil count at the time of diagnosis was significantly higher in patients with persistent valvular involvement during follow-up. ROC curve analysis further supported this observation and demonstrated that neutrophil count and NLR had moderate predictive value for the development of RHD.

We also observed a positive correlation between NLR and CRP levels, suggesting that NLR may reflect systemic inflammatory activity in patients with ARF [9,26-30].

From a clinical perspective, simple inflammatory markers derived from routine laboratory tests may provide additional support in the early evaluation of patients with ARF. The NLR, which can be calculated from a standard complete blood count, has the advantages of being inexpensive, readily available, and rapidly obtainable in most healthcare settings [9,26,29,30].

## Conclusions

In conclusion, our findings suggest that platelet indices, such as MPV and P-LCR, are not significantly associated with ARF. However, neutrophil count and NLR appear to reflect inflammatory activity in the disease. Furthermore, neutrophil count at the time of diagnosis may help identify patients at risk for developing RHD, and ROC analysis demonstrated a moderate predictive value for these parameters. These findings should be considered preliminary and hypothesis-generating, and further prospective studies are needed to confirm these results.
